# Appetite hormones rather than proinflammatory cytokines differentiate bipolar I and II depression: a classification and regression tree analysis

**DOI:** 10.1007/s00406-025-02132-7

**Published:** 2025-10-30

**Authors:** Shao-Lun Ko, Ya-Mei Bai, Ju-Wei Hsu, Shih-Jen Tsai, Mu-Hong Chen

**Affiliations:** 1 Department of Psychiatry, Taipei Veterans General Hospital, No. 201, Shih-Pai Road, Sec. 2, Taipei, 11217 Taiwan; 2 Department of Psychiatry, College of Medicine, National Yang Ming Chiao Tung University, Taipei, Taiwan; 3 Institute of Brain Science, National Yang Ming Chiao Tung University, Taipei, Taiwan; 4 School of Medicine, National Yang Ming Chiao Tung University, Taipei, Taiwan; 5 Department of Medical Education, Taipei Veterans General Hospital, Taipei, Taiwan

**Keywords:** Classification and regression tree, Bipolar disorder, Appetite hormones, Cytokines

## Abstract

**Background:**

Appetite hormones and proinflammatory cytokines play a role in differentiating between bipolar I disorder (BD1) and bipolar II disorder (BD2). In this study, we developed a composite predictor of appetite hormones and proinflammatory cytokines to differentiate between BD1 and BD2.

**Methods:**

Adult patients aged 20–59 with either BD1 or BD2 and experiencing a major depressive episode were included in the study. Cytokines such as C-reactive protein, interleukin-2, interleukin-6, and tumor necrosis factor-α and appetite hormones such as leptin, adiponectin, ghrelin, and insulin were evaluated as potential predictors through a classification and regression tree (CRT) to differentiate between BD1 and BD2.

**Results:**

A composite predictor of adiponectin, leptin, and ghrelin was significantly more accurate (for BD1: area under the curve = 0.897; for BD2: area under the curve = 0.905, *P* > 0.05) in differentiating between BD2 and BD1 than any single predictor (four appetite hormones and six cytokines). High levels of adiponectin and ghrelin and high and low levels of leptin (≤ 4430.8 and > 10,957.2 ng/L) were associated with BD2, whereas low levels of adiponectin and ghrelin and intermediate levels of leptin were associated with BD1.

**Conclusions:**

The composite predictor of appetite hormones showed potential for distinguishing between BD1 and BD2 during depressive episodes. However, given the exploratory nature of the analysis and the limited sample size, further studies are needed to validate the model’s utility in clinical settings and to better understand the pathomechanisms underlying BD subtypes

## Introduction

Bipolar disorder (BD) is a chronic psychiatric condition characterized by recurrent mood episodes, affecting approximately 5% of the global population [1]. Bipolar I disorder (BD1) involves pronounced manic and depressive episodes, whereas bipolar II disorder (BD2) primarily involves alternating episodes of depression and hypomania [2]. According to the *Diagnostic and Statistical Manual of Mental Disorders*,* Fifth Edition* (DSM-5), BD1 and BD2 share the same criteria for major depressive episodes. Nevertheless, the clinical threshold for mania and hypomania is based on the duration of affective episodes and levels of individual functioning and may be ambiguous in actual clinical settings [3]. BD2 was officially recognized only 30 years ago in the DSM diagnostic system [3]. Some psychiatrists argue that combining BD1 and BD2 into a unified category may provide consistency in clinical management and promote the concept of a bipolar spectrum that includes diverse clinical manifestations of bipolarity. Nevertheless, no clear biological differences have yet been identified between the two conditions [4].

Emerging evidence has indicated clinical differences between BD1 and BD2 [4–9], underscoring the importance of differentiating between them in clinical practice, with BD1 commonly presenting severe manic or psychotic symptoms, whereas BD2 dominantly presents recurrent or chronic depressive symptoms [5]. A study involving 41 adolescents with BD1 and 68 with BD2 demonstrated that BD1 was associated with greater functional impairment, psychotic features, and higher rates of psychiatric hospitalization compared with BD2 [5]. Similarly, a study of young individuals aged 8–18 years reported that patients with BD1 had more inpatient admissions than those with BD2, although both subtypes had similar rates of suicide attempts [6]. A recent systematic review further reported that psychotic symptoms are approximately twice as common in BD1 than BD2 and indicated that BD1 is consistently associated with greater illness severity, including poorer insight and greater cognitive impairment [7].

Previous studies have indicated the involvement of appetite hormones, such as leptin, insulin, adiponectin, and ghrelin, in the pathophysiology of BD. Cordas et al. reported that patients with bipolar depression exhibited lower leptin levels compared with patients with major depressive disorder (MDD) and healthy controls [10]. Hsu et al. discovered that patients with BD who were in a relatively stable state (Clinical Global Impression–Severity score ≤ 4) exhibited higher insulin levels and lower leptin levels compared with healthy individuals [11]. A study involving 33 patients with bipolar depression and 66 with MDD revealed that a composite predictor of ghrelin and tumor necrosis factor-α (TNF-α) presumably differentiated between bipolar depression and MDD [12]. Misiak et al. reported that the levels of leptin and insulin were considerably higher in patients with BD during the euthymic state compared with healthy individuals [13]. They further indicated that the adiponectin dysregulation was specific to BD1, suggesting a potential role of appetite hormones in differentiating between BD subtypes [13].

Evidence has shown that proinflammatory and anti-inflammatory cytokine dysregulation plays a key role in the pathophysiology of BD [14]. Solmi et al. discovered that the levels of C-reactive protein (CRP), interleukin-6 (IL-6), and TNF-α were higher in patients with BD than in the control group [15]. They also reported that the levels of CRP and TNF-α increased during both depressive and manic episodes, but not during the euthymic state, whereas the levels of IL-6 remained high regardless of the individual’s mood state [15]. Previous studies have indicated that levels of peripheral proinflammatory cytokines can serve as biomarkers for differentiating between BD1 and BD2. For instance, in a study involving 234 patients with BD1, 260 patients with BD2, and 140 healthy controls, Wang et al. discovered that patients with BD1 exhibited higher levels of IL-8 compared with patients with BD2 [16]. In another study, Bai et al. reported higher levels of IL-2 in patients with BD1 than in those with BD2 [17]. By contrast, Hua et al. reported significantly higher levels of TNF-α in patients with BD1 and BD2 compared with the control group, but these levels did not differ between patients with BD1 and BD2 [18].

In this study, given the major role of appetite hormone and cytokine dysregulation in the pathomechanisms of BD, we applied a classification and regression tree (CRT) method as an exploratory, data-driven approach to identify potential biomarker combinations that may differentiate between BD subtypes during major depressive episodes. We also examined whether the hypothesized composite profiles identified by CRT offered greater discriminatory potential than any individual biomarker alone.

## Methods

### Participants

Adult patients aged between 20 and 59 years who were diagnosed with BD1 or BD2 by board-certified psychiatrists using the Structured Clinical Interview for DSM-5 (SCID-5), based on the Diagnostic and Statistical Manual of Mental Disorders, Fifth Edition (DSM-5), and were in a major depressive episode were enrolled in the current study as the study groups. Age- and sex-matched healthy controls who did not have a DSM-5 diagnosis; who were not pregnant or breastfeeding; and who did not have a severe physical disease (i.e., epilepsy, stroke, or systemic autoimmune diseases) or unstable physical illnesses were recruited from the community via the advertisement as the control group. For all participants, depressive symptoms were assessed by Montgomery–Åsberg Depression Rating Scale (MADRS). This study was approved by the Institutional Review Board of Taipei Veterans General Hospital and conducted in accordance with the Declaration of Helsinki. Written informed consent was obtained from all participants prior to their enrollment in the study.

### Measurement of pro-inflammatory cytokines and appetite hormones

Fasting serum samples were collected in serum separator tubes, clotted for 30 min, and stored at − 80 °C until use. Pro-inflammatory cytokines, including IL-2, IL-6, TNF-α, CRP, P-selectin, and monocyte chemoattractant protein (MCP)-1, were assayed using enzyme-linked immunosorbent assay (ELISA) kits (R&D systems, Minneapolis, MN, USA) for all participants. The appetite hormones, including leptin, ghrelin, insulin, adiponectin, were examined. Ghrelin was measured using a radioimmunoassay (RIA) kit (Peninsula Laboratories, Inc., San Carlos, CA, USA). Insulin concentrations were analyzed using a radioimmunoassay kit (Coat-A Count Insulin; Diagnostic Product Corporation, Los Angeles, CA, USA). Serum adiponectin level was measured using a quantitative Human Adiponectin ELISA Kit (B-Bridge International, Inc., Mountain View, CA, USA). All assays were performed according to the vendor’s instructions. The final absorbance of each sample of the mixture was measured and analyzed at 450 nm using an ELISA plate reader with Bio-Tek Power Wave Xs and Bio-Tek’s KC junior software (Winooski, VT, USA). The standard range was considered as specified in the vendor’s instructions. A linear regression R-square value of at least 0.95 was considered a reliable standard curve.

### Statistical Analysis

CRT analysis was employed to explore potential interactions among predictors and to model nonlinear relationships in an interpretable way [19, 20]. When compared with other complex modelling techniques, CRT analysis requires small sample sizes (10 events per variable) to obtain a reasonably predictive modelling with stable performance [21]. While CRT does not rely on parametric assumptions, it involves model-building decisions, including the choice of splitting criteria and stopping rules [22]. In this study, all variables of interest were entered into the model without prior univariate screening. These included the total score of MADRS, BMI, levels of pro-inflammatory cytokines (IL-2, IL-6, TNF-α, CRP, P-selectin, MCP-1), and appetite hormones (leptin, ghrelin, insulin, adiponectin). CRT constructs a decision tree using a series of binary splits to maximize the sensitivity and specificity of the classification [22], aiming to distinguish individuals with BD1, BD2, or those with no diagnosis. Tree pruning was performed to minimize misclassification and reduce overfitting [22]. The maximum tree depth was set to four levels, with a minimum of 20 cases per parent node and 10 per child node. Model performance was assessed using gains and risk charts, which provide the proportion of target categories (BD1 vs. BD2 vs. control) in each node. The k-fold cross-validation method (k = 10) was employed to validate the classification of the decision tree and assess the model’s stability [23]. Model performance was further evaluated using receiver operating characteristic (ROC) curve analysis. All statistical analyses were conducted using SPSS (Version 22.0), with two-tailed significance set at *p* < 0.05.

## Results

This study included 145 subjects, including 44 patients with BD1, 51 with BD2, and 50 healthy controls (Table 1). The demographic and clinical characteristics of the three groups did not differ for age, sex, or levels of leptin, adiponectin, CRP, IL-2, IL-6, or P-selectin (Table 1). Compared with patients with BD2, patients with BD1 demonstrated significantly higher levels of insulin and TNF-α, significantly lower levels of ghrelin, but similar BMI and MCP-1 levels (Table 1).

**Table 1:** Demographic data and levels of appetite hormones and pro-inflammatory cytokines among patients with BD1 and BD2 and the controls

	A. BD1 (*n* = 44)	B. BD2 (*n* = 51)	C. Control (*n* = 50)	*p*-value	post-hoc analysis
Age (years)	37.77 (10.77)	36.67 (9.86)	35.56 (8.84)	0.553	
Sex^*^				0.389	
Male	30 (68.2)	37 (72.5)	30 (60.0)		
Female	14 (31.8)	14 (27.5)	20 (40.0)		
MADRS (SD)	19.36 (6.35)	18.92 (6.28)	-	0.734	
YMRS (SD)	3.52 (2.57)	4.39 (2.59)		0.105	
Duration of illness (years, SD)	9.24 (9.28)	6.82 (5.95)		0.129	
BMI (kg/m^2^)	25.21 (5.52)	23.55 (4.68)	23.02 (2.87)	0.051	A > C
Appetite hormones					
Leptin (ng/L)	11195.10 (9709.39)	9395.38 (8505.70)	12730.70 (12934.74)	0.287	
Adiponectin (ug/L)	7154.82 (6042.90)	6588.27 (4558.79)	5312.76 (6092.47)	0.259	
Ghrelin (fmol/mL)	216.28 (230.27)	323.43 (300.97)	65.85 (80.58)	< 0.001	B > A > C
Insulin (mU/L)	9.83 (15.48)	5.63 (7.11)	5.00 (6.24)	0.051	A > B ~ C
Proinflammatory cytokines					
CRP (ug/L)	1688.29 (1962.38)	1548.36 (1820.77)	996.58 (1091.47)	0.099	
IL-2 (pg/mL)	702.11 (271.03)	617.93 (243.12)	558.90 (225.75)	0.076	
IL-6 (pg/L)	34007.23 (11963.65)	34122.77 (10256.09)	36561.92 (6771.74)	0.679	
TNF-α (pg/L)	1062.90 (304.79)	931.02 (244.85)	860.37 (142.98)	< 0.001	A > B ~ C
P-selectin (ng/mL)	77.40 (41.19)	81.63 (37.91)	87.58 (32.62)	0.413	
MCP-1 (pg/mL)	260.79 (103.97)	252.86 (84.75)	301.33 (96.35)	0.026	C > A ~ B

Data were presented as mean (standard deviation) or count (percentage)^*^

Abbreviation: BD: bipolar disorder; BMI: body mass index; TNF-α: tumor necrosis factor-α; IL: interleukin; CRP: C-reactive protein; MCP: monocyte chemoattractant protein; MADRS: Montgomery-Asberg Depression Rating Scale

**Fig. 1:**
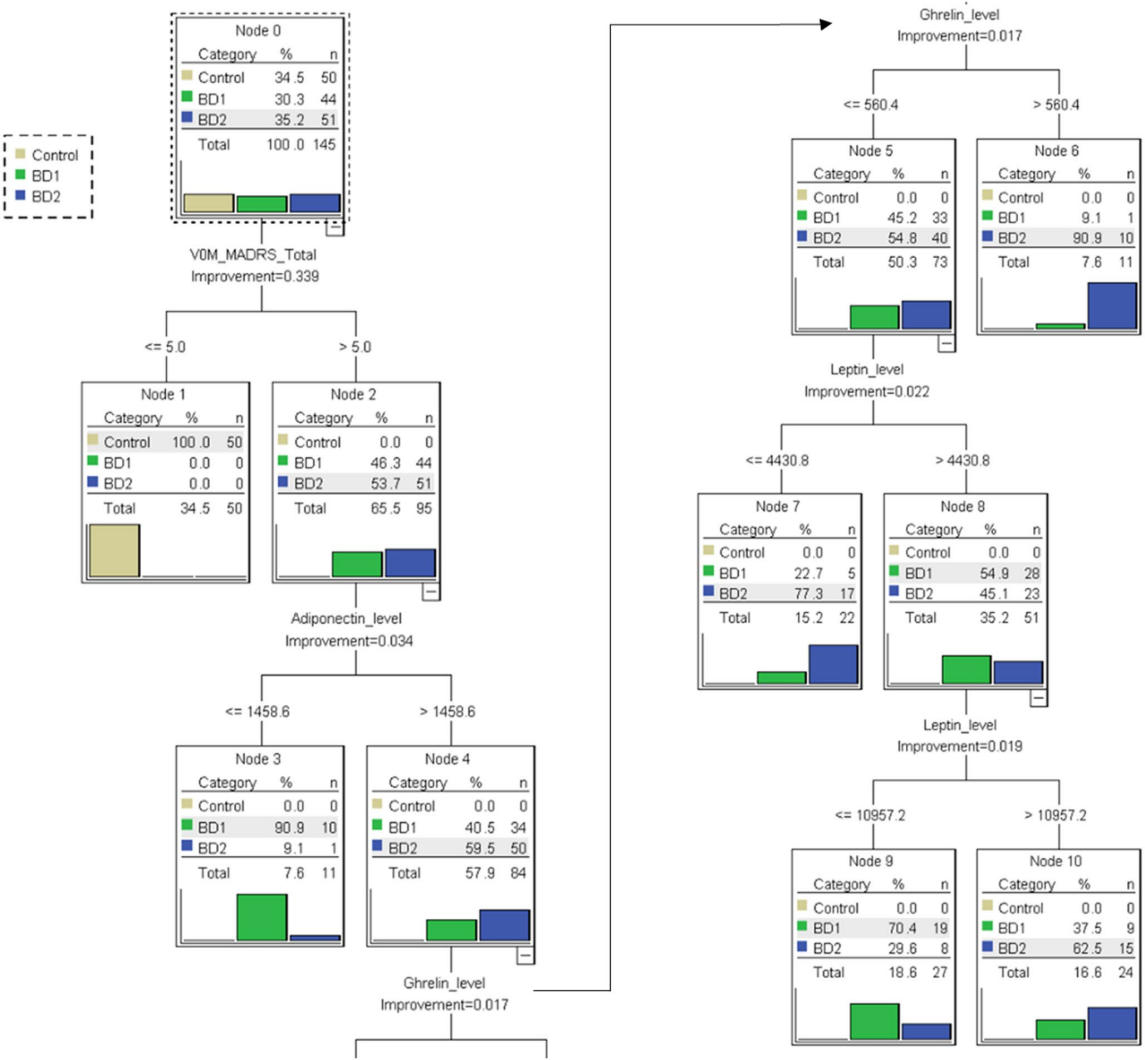
Classification and regression tree for the differentiation of BD1 and BD2

The decision tree analysis indicated that among patients with BD, the strongest predictor for differentiating BD1 from BD2 was the adiponectin level (Fig. 1, nodes 3 and 4). Compared with patients with low adiponectin levels (≤ 1458.6 ug/L), those with high adiponectin levels (> 1458.6 ug/L) were more likely to be diagnosed as having BD2 (59.5% vs. 9.1% for patients with high adiponectin levels and those with low adiponectin levels, respectively). Among the patients with high adiponectin levels (> 1458.6 ug/L), the ghrelin level was the most prominent predictor (Fig. 1, nodes 5 and 6). Patients with high ghrelin levels (> 560.4 fmol/mL, 90.9%) were more likely to have BD2 than those with low ghrelin levels (≤ 560.4 fmol/mL, 54.8%). Among patients with high adiponectin levels (> 1458.6 ug/L) and low ghrelin levels (≤ 560.4 fmol/mL), the leptin level was the most prominent predictor (Fig. 1, nodes 7, 8, 9, and 10). Patients with BD1 were more likely to have a leptin level between 4430.8 and 10957.2 ng/L. In contrast, leptin levels on both ends (≤ 4430.8 and > 10957.2 ng/L) were associated with BD2.

The gain chart demonstrates the predictive power of each model for the target category (Table 2). Nodes were arranged in descending order of gain score. In the current study, the node with the highest gain score for BD1 was node 3, which designated patients with adiponectin levels of ≤ 1458.6 ug/L (Fig. 1; Table 2). Conversely, the node with the highest gain score for BD2 was node 6, which indicated patients with adiponectin levels of > 1458.6 ug/L and ghrelin levels of > 560.4 fmol/mL (Fig. 1; Table 2).

**Table 2:** Gain index of the classification of BD1 and BD2

		Node-by-Node						Cumulative					
Node	Markers	Node		Gain				Node		Gain			
		N	Percent	N	Percent	Response	Index	N	Percent	N	Percent	Response	Index
		BD1											
3	Adiponectin ≤ 1458.6	11	7.6%	10	22.7%	90.9%	299.6%	11	7.6%	10	22.7%	90.9%	299.6%
9	Leptin ≤ 10957.2	27	18.6%	19	43.2%	70.4%	231.9%	38	26.2%	29	65.9%	76.3%	251.5%
10	Leptin > 10957.2	24	16.6%	9	20.5%	37.5%	123.6%	62	42.8%	38	86.4%	61.3%	202.0%
7	Leptin ≤ 4430.8	22	15.2%	5	11.4%	22.7%	74.9%	84	57.9%	43	97.7%	51.2%	168.7%
6	Ghrelin > 560.4	11	7.6%	1	2.3%	9.1%	30.0%	95	65.5%	44	100.0%	46.3%	152.6%
1	MADRS ≤ 5	50	34.5%	0	0.0%	0.0%	0.0%	145	100.0%	44	100.0%	30.3%	100.0%
		BD2											
6	Ghrelin > 560.4	11	7.6%	10	19.6%	90.9%	258.5%	11	7.6%	10	19.6%	90.9%	258.5%
7	Leptin ≤ 4430.8	22	15.2%	17	33.3%	77.3%	219.7%	33	22.8%	27	52.9%	81.8%	232.6%
10	Leptin > 10957.2	24	16.6%	15	29.4%	62.5%	177.7%	57	39.3%	42	82.4%	73.7%	209.5%
9	Leptin ≤ 10957.2	27	18.6%	8	15.7%	29.6%	84.2%	84	57.9%	50	98.0%	59.5%	169.2%
3	Adiponectin ≤ 1458.6	11	7.6%	1	2.0%	9.1%	25.8%	95	65.5%	51	100.0%	53.7%	152.6%
1	MADRS ≤ 5	50	34.5%	0	0.0%	0.0%	0.0%	145	100.0%	51	100.0%	35.2%	100.0%

Abbreviation: BD: bipolar disorder; TNF-alpha: tumor necrosis factor-α; MADRS: Montgomery-Asberg Depression Rating Scale

BD: bipolar disorder; TNF-alpha: tumor necrosis factor-α; MADRS: Montgomery-Asberg Depression Rating Scale

BD: bipolar disorder; BMI: body mass index; TNF-alpha: tumor necrosis factor-α; IL: interleukin; CRP: C-reactive protein; MCP: monocyte chemoattractant protein; MADRS: Montgomery-Asberg Depression Rating Scale; AUC, area under curve

**Fig. 2:**
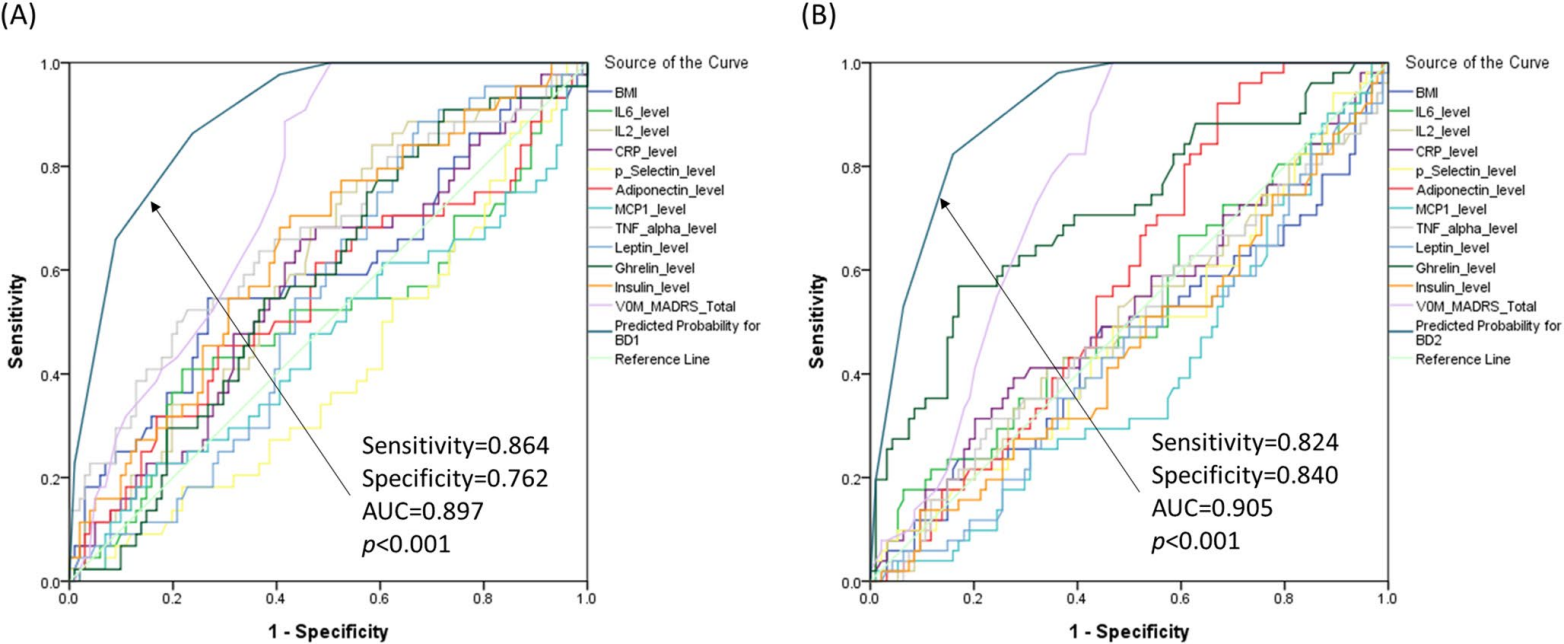
Receiver operating characteristic curve of the predicted probability for (A) BD1 and (B) BD2

The ROC curve analysis evaluated the CRT-derived composite predictor of adiponectin, leptin, and ghrelin levels for distinguishing each bipolar subtype from all other groups. For distinguishing BD1 from BD2 and healthy controls, the area under the ROC curve (AUC) was 0.897 (95% CI: 0.847–0.947; *p* < 0.001). For distinguishing BD2 from BD1 and healthy controls, the AUC was 0.905 (95% CI: 0.858–0.952; *p* < 0.001). In both analyses, the composite model outperformed individual clinical and demographic factors (all *p* > 0.05; Fig. 2). Results of the k-fold cross-validation test indicated the stability of the model. Risk estimates (0.548) and standard errors (0.058) of the model were not different from the results of the model without a cross-validation test.

## Discussion

In this study, we used CRT to identify potential biomarker combinations that may differentiate between BD subtypes during major depressive episodes. Our findings suggested that a composite predictor of appetite hormones, including adiponectin, ghrelin, and leptin, may differentiate between BD1 and BD2. Patients with BD1 tended to exhibit lower levels of ghrelin and adiponectin, with intermediate levels of leptin, whereas patients with BD2 tended to exhibit higher levels of adiponectin and ghrelin, with both high and low levels of leptin. In other words, the predictive power of our composite predictor was considerably higher than that of any single predictor (each appetite hormone and cytokine). However, the current results were exploratory and hypothesis-generating, rather than confirmatory or diagnostic.

Overall, our findings indicate that our composite predictor of appetite hormones (leptin, ghrelin, and adiponectin) has greater predictive power than any single biomarker, including proinflammatory cytokines. Previous studies have indicated certain disparities in the levels of proinflammatory cytokines between BD1 and BD2 [16, 17]. As mentioned, Bai et al. discovered that patients with BD2 exhibited significantly lower levels of TNF-α compared with those with BD1 [17]. Wang et al. reported that patients with BD1 exhibited significantly higher levels of IL-8 compared with those with BD2. However, they discovered no significant differences in the levels of TNF-α, CRP, and transforming growth factor-β1 between the two cohorts [16]. In this CRT-based study, we discovered that proinflammatory cytokines did not play a role in differentiating between BD1 and BD2 during major depressive episodes. Further research is required to determine whether proinflammatory cytokines may serve as potential biomarkers to differentiate between BD1 and BD2.

Adiponectin, the most abundant adipokine secreted by adipose tissue, plays a key role in various physiological processes, including energy metabolism, inflammation, and vascular physiology [24]. In this study, we discovered that adiponectin levels were lower in patients with BD1 than in those with BD2, inconsistent with the findings of Misiak et al., who indicated a positive correlation between adiponectin levels and BD1 [13]. Misiak et al.’s finding was based on meta-regression of only seven studies that reported the proportion of BD subtypes and not direct comparisons between patients with BD1 and BD2 [13]. Additionally, high heterogeneity (I² >95% in most analyses) and potential publication bias (Egger’s test *p* = 0.007) were noted [13]. In addition, Rosso et al. examined potential alterations in markers of metabolic dysfunction, including ghrelin, leptin, and insulin, but identified no significant differences between different subtypes [25]. The discrepancy of the current study with the findings of Rosso et al. may relate to differences in illness stage. The mean age of their sample was 50.2 years, likely reflecting later-stage BD, where the cumulative burden of the illness effect may contribute to more prominent hormone dysregulation [25]. Our younger sample (mean age: 37.77 for BD-I, 36.67 for BD-II) may represent earlier-stage BD, where subtype-specific hormonal differences were more detectable. Furthermore, multiple studies have examined the anti-inflammatory role of adiponectin [24], suggesting a correlation between a strong inflammatory state and BD1 [16, 17]. Overall, our findings of reduced adiponectin levels in BD1 may support the notion of a stronger inflammatory state in BD1 than in BD2 [14, 16, 17]. Adiponectin is characterized by antiapoptotic and antioxidant effects, contributing to its cardioprotective mechanism [24]. Therefore, reduced adiponectin levels in BD1 may partially explain the findings of Fiedorowicz et al., who reported that patients with BD1 exhibited a higher risk of cardiovascular mortality compared with those with BD2 [26].

Ghrelin is a gastric acylated peptide that increases body weight and induces adiposity by acting on the hypothalamic melanocortinergic system [27]. Multiple studies have indicated that ghrelin has a protective effect on cognitive function and plays a key role in hippocampal neurogenesis, thus enhancing cognitive function [28–31]. For instance, Li et al. discovered that ghrelin improved cognition in a depression mouse model [29]. Sassi et al. reported that a decrease in the ratio of plasma acylated ghrelin (AG) to unacylated ghrelin (UAG) was presumably associated with cognitive impairments in patients with Parkinson’s disease, with AG being the active form of circulating ghrelin and UAG being its precursor [30]. In a previous study, we discovered a positive correlation between ghrelin levels and executive functioning in patients with bipolar depression [31]. Multiple studies have indicated that patients with BD1 typically experience greater cognitive deficits compared with those with BD2 [5, 14, 18], consistent with our findings of ghrelin level as a potential biomarker differentiating between BD2 (higher ghrelin levels) and BD1 (lower ghrelin levels).

Leptin is a protein predominantly secreted by adipocytes; its plasma levels are strongly correlated with body fat mass [32], leading to a decrease in body weight [33]. In this study, we discovered that BD2 was associated with high and low levels of leptin, whereas BD1 was associated with intermediate levels of leptin. Previous research into leptin’s involvement in BD has yielded inconsistent findings, with reports of both high and low levels of leptin in patients with bipolar depression [10, 34], which may partially explain our observation of varied leptin distributions between BD1 and BD2. Factors such as higher depressive predominant polarity, atypical depression, rapid cycling, and prevalence of obesity in BD2 than in BD1 may explain why we identified a subgroup of patients with BD2 with high leptin levels [35, 36]. By contrast, the other subgroup of patients with BD2 exhibited low leptin levels, suggesting a more variable nature of leptin levels in patients with BD2 than in those with BD1. Further research is required to determine whether leptin levels can be used as a reliable biomarker of dysregulation in mood, appetite, and body weight, which may be specific to BD2.

Finally, given adiponectin with an anti-inflammatory effect and ghrelin with a precognitive effect [24, 27], our findings of the lower levels of adiponectin and ghrelin in BD1 than in BD2 may echo the evidence that BD1 was in a severer inflammatory state and exhibited greater cognitive impairment compared with BD2 [5, 14, 16–18]. In order to reduce the complications from the chronic inflammation and cognitive impairment, the anti-inflammatory and precognitive treatment may be initiated earlier for patients with BD1 than those with BD2 [37].

This study has some limitations. First, the cross-sectional design of this study precluded any inference that appetite hormones, cytokines, and BD are causally related to each other. Second, patients with BD remained on their medications throughout the study, indicating that these medications presumably affected their levels of inflammatory cytokines and appetite hormones [38–42]. Although allowing patients to continue their medications during clinical studies is a more ethical procedure, drug-free studies may be required to validate our findings. Third, the present study only focused on depressive states of BD1 and BD2. Further investigation is necessary to determine whether our findings may apply to euthymic or manic states.

In conclusion, the exploratory and hypothesis-generating findings of our study suggested that a composite predictor of the fasting levels of adiponectin, ghrelin, and leptin driven by CRT may potentially aid in the differentiation between BD1 and BD2. High levels of adiponectin and ghrelin and both high and low levels of leptin are associated with BD2, whereas low levels of adiponectin and ghrelin and intermediate levels of leptin are associated with BD1. The predictive performance of a composite predictor is higher that of any single predictor. Further clinical research is required to validate our findings and examine the diverse pathomechanisms underlying BD1 and BD2.

## Data Availability

Due to the nature of this research, participants of this study did not agree for their data to be shared publicly. The datasets generated during and/or analyzed during the current study are available from the corresponding author on reasonable request.
